# Single Seed Near-Infrared Hyperspectral Imaging for Classification of Perennial Ryegrass Seed

**DOI:** 10.3390/s23041820

**Published:** 2023-02-06

**Authors:** Priyanka Reddy, Joe Panozzo, Kathryn M. Guthridge, German C. Spangenberg, Simone J. Rochfort

**Affiliations:** 1 Agriculture Victoria, AgriBio, Centre for AgriBioscience, Bundoora, VIC 3083, Australia; 2 School of Applied Systems Biology, La Trobe University, Bundoora, VIC 3083, Australia

**Keywords:** phenotyping, PLS-DA, ANN-DA, SVM, genetic algorithm, endophyte, chemical imaging, NEA12, *Epichlo*ë

## Abstract

The detection of beneficial microbes living within perennial ryegrass seed causing no apparent defects is challenging, even with the most sensitive and conventional methods, such as DNA genotyping. Using a near-infrared hyperspectral imaging system (NIR-HSI), we were able to discriminate not only the presence of the commercial NEA12 fungal endophyte strain but perennial ryegrass cultivars of diverse seed age and batch. A total of 288 wavebands were extracted for individual seeds from hyperspectral images. The optimal pre-processing methods investigated yielded the best partial least squares discriminant analysis (PLS-DA) classification model to discriminate NEA12 and without endophyte (WE) perennial ryegrass seed with a classification accuracy of 89%. Effective wavelength (EW) selection based on GA-PLS-DA resulted in the selection of 75 wavebands yielding 88.3% discrimination accuracy using PLS-DA. For cultivar identification, the artificial neural network discriminant analysis (ANN-DA) was the best-performing classification model, resulting in >90% classification accuracy for Trojan, Alto, Rohan, Governor and Bronsyn. EW selection using GA-PLS-DA resulted in 87 wavebands, and the PLS-DA model performed the best, with no extensive compromise in performance, resulting in >89.1% accuracy. The study demonstrates the use of NIR-HSI reflectance data to discriminate, for the first time, an associated beneficial fungal endophyte and five cultivars of perennial ryegrass seed, irrespective of seed age and batch. Furthermore, the negligible effects on the classification errors using EW selection improve the capability and deployment of optimized methods for real-time analysis, such as the use of low-cost multispectral sensors for single seed analysis and automated seed sorting devices.

## 1. Introduction

Perennial ryegrass (*Lolium perenne* L.) is used for forage and turf in temperate regions throughout the world, including Northern Europe, the Pacific Northwest of the USA, Japan, South-Eastern Australia and New Zealand [1]. It is the most commonly utilised pasture grass on dairy farms in Australia and has high economic importance. The pasture species typically forms a symbiotic relationship with a naturally occurring asexual *Epichloe* spp. fungal endophyte (henceforth referred to as endophytes). The endophyte confers unique properties that have led to its utilization as a trait in cultivar development, in particular for the biocontrol of insect pests, enhancing pasture performance and persistence via the production of compounds that confer resistance to biotic and abiotic stresses.

Cultivar distinctness and purity, as well as physiological characteristics, are hallmarks of seed quality [2,3]. In the plant breeding program, determining distinctness, uniformity and stability (DUS) traits through consecutive generations is critical for commercial rights over new plant varieties and the development of new cultivars in the market. In pasture grasses, such as ryegrass, varietal purity extends to endophyte content and purity; thus, batch purity of high-quality cultivars is important in marketed forage species. While there are established quality control measures for cultivar discrimination [4] and endophyte content and purity [5], using sensitive tests such as DNA-based genotyping is often complex, labour intensive, expensive and destructive, thus an evaluation is limited to a randomly selected subset of seeds from a batch and not all batches are tested [6]. These attributes are not ideal when seeds are valuable and/or the supply is limited.

The well-established non-destructive and high-throughput technique, near-infrared spectroscopy (NIRS), has been used since the 1960s for quality assessment of agricultural products [7,8]. NIRS for seed evaluation typically involves the generation of an average spectrum for a set of seeds or individual seeds from only a few selected points across the sample. With the development of NIRS coupled with imaging techniques in the late 1990s, the capability has tremendously improved as both spatial and spectral data can be acquired for samples [9]. The spatial resolution indicates the distribution of the chemical constituents within a sample and can vary depending on the size of the pixel, which influences the signal strength. With the advantages of being a non-destructive and potentially high-throughput technique for the acquisition of both qualitative and quantitative data, NIR-HSI investigations for seed quality assessments have become increasingly popular. As reviewed by Reddy et al. [6], NIR-HSI has been applied in the varietal classification of plant species, such as cotton and maize, as well as the detection of disease-causing fungal pathogens on seed [10,11,12,13,14,15,16]. However, the potential of the technology for use in seed-based cultivar discrimination in pastures, such as perennial ryegrass and, importantly, beneficial endophyte detection, has not been explored.

There is good evidence in the literature that varietal identification can be achieved with at least 80% accuracy [6]. Zhu et al. [17] identified seven varieties of cotton seeds using NIR-HSI wavelengths 942–1646 nm. Classification models, including partial least squares discriminant analysis (PLS-DA), logistic regression (LR) and support vector machine (SVM), were applied to full wavelengths as well as effective wavelengths (EW) that were selected according to principal component analysis (PCA) loadings. PLSDA, LR and SVM models were also used as classifiers for deep learning architectures. Zhao et al. [18] identified three grape seed varieties using NIR-HSI in the spectral range 874–1734 nm. An SVM model was built for the classification using effective wavelengths selected by PCA loadings. Kong et al. [19] identified four rice seed cultivars using NIR-HSI wavelengths covering 874–1734 nm. PLS-DA, soft independent modelling of class analogy (SIMCA), k-nearest neighbour (KNN) and support vector machine (SVM) and random forest (RF) were applied. EW was selected using weighted regression coefficients of the PLS-DA. The 12 optimal wavelengths were used to develop PLS-DA, KNN, SVM and RF models. Thus, NIR-HSI has been successfully used for varietal classification for many agriculturally relevant seeds, and while no literature currently exists on NIR-HSI chemical imaging to identify pasture seed varieties, the successful application of the technology using seed from other species suggests it is feasible. 

The presence of the naturally occurring, asymptomatic, fungal symbiont in perennial ryegrass contributes to the complexity of the morphology of the seed and its classification. While there is no literature describing the use of NIR-HSI for the detection of beneficial fungal endophytes in seed, previous reports investigating fungal phytopathogens in seed have achieved similar accuracies to varietal identifications of at least 80%. Kheiralipour et al. [10] identified aflatoxin contamination of pistachio kernels at different growth stages using NIR-HSI wavelengths 900–1700 nm. Classification models were developed using linear discriminant analysis (LDA) and quadratic discriminant analysis (QDA) based on EW selected from PCA loadings. Fungal infections were identified in canola seeds using NIR-HSI covering wavelengths 1000–1600 nm [11]. LDA, QDA and Mahalanobis discriminant classifiers were applied to effective wavelengths selected based on PCA loadings. 

These studies present promising outputs in calibration, validation and prediction, and the utilization of NIR-HSI is becoming a necessary advancement for real-time analysis of individual seed quality parameters. These outcomes are being recognized by organisations that develop standard methods for seed quality, such as the International Seed Testing Association (ISTA) and the Association of Official Seed Analysts (AOSA) [20]. Although seed phenotyping is more challenging, concerted and ongoing research and development will lead to the industry uptake of routine and real-time applications. 

The objective of the present study was to detect, for the first time, the presence/absence of the beneficial NEA12 fungal endophyte as well as discriminate cultivars of perennial ryegrass seed within the same cohort, irrespective of seed age and batch. The new means to phenotype based on established NIR-HSI imaging pipeline techniques would allow for the development of a real-time high-throughput seed phenotyping tool for seed sorting.

## 2. Materials and Methods

### 2.1. Sample Preparation

Cultivars of perennial ryegrass seed were obtained from Barenbrug, Christchurch, New Zealand. The total number of samples (*n*) used in this study was 1057. The sample population of NEA12 (*n* = 577) endophyte-infected seed (henceforth referred to as E+) consisted of four cultivars, including Alto (*n* = 96; received 11 July 2018); Trojan (*n* = 193; received 11 July 2018); Rohan (*n* = 96; received 1 September 2017 and *n* = 96; received 17 December 2013); Governor (*n* = 96; received 1 September 2017). The seed samples without endophyte (WE) (*n* = 480) (henceforth referred to as E−) also consisted of four cultivars, including Bronsyn (*n* = 96; received 11 July 2018); Trojan (*n* = 96; received 11 July 2018 and *n* = 96; received 17 December 2013); Rohan (*n* = 96; received 17 December 2013); Governor (*n* = 96; received 17 December 2013).

All seeds were stored in a controlled environment room (CER) at 4 °C.

### 2.2. Hyperspectral Imaging System

This system consisted of a short-wave infrared (SWIR) hyperspectral camera (Specim Finland), comprising a cryogenically cooled MCT detector scanning 288 spectral bands between 1000–2500 nm with 384 spatial pixels and 24 × 24 μm pixel size. The size of an individual uncompressed raw image file of 96 seeds was approximately 217 MB.

### 2.3. Image Acquisition and Normalization

Perennial ryegrass seeds were arranged on a black plate and placed on a Specim SisuCHEMA imaging analyser (Specim Finland) at a scan rate of 3 mm.s^−1^. Samples were scanned in an airconditioned laboratory with temperature and humidity maintained at approximately 22–25 °C and 30–40% relative humidity (RH), respectively. Seeds were stored in the laboratory for 24 h prior to scanning to allow seed temperature to equilibrate to the ambient environment. A hyperspectral image was formed by 288 congruent grayscales sub-images representing the intensities of 288 wavelength bands. Thus, 3D hypercube data, representing the hyperspectral images, contained the spectral and spatial information used to identify perennial ryegrass seed cultivars and endophyte presence. Before hyperspectral image acquisition, white and dark reference images were acquired. The acquisition of the dark reference image is to remove the influence of dark current in the camera. A dark current is a current or flow of electrons generated even when no photons are incident on the camera. The valence electrons are thermally excited within the silicon chip comprising the charged couple device (CCD) and into the conduction band. The dark reference image was acquired by turning off the light source together with covering the camera lens completely with its opaque cap, while the white reference image was acquired by using a white Teflon tile with nearly 100% reflectance.

Then the calibrated image or reflectance value (*R*) was calculated by using the raw hyperspectral image (*I_S_*), white reference image (*I_W_*) and dark reference image (*I_D_*) (Equation (1)) [21]:

(1) $$R=\frac{I_{S}-I_{D}}{I_{W}-I_{D}}$$

After the hyperspectral images were normalized, the seeds were corrected based on (Equation (1)). The average spectrum of all pixels in each seed was used as the spectrum of the sample. In total, 288 spectra for each individual seed were acquired.

### 2.4. Image Processing

**Figure 1:**
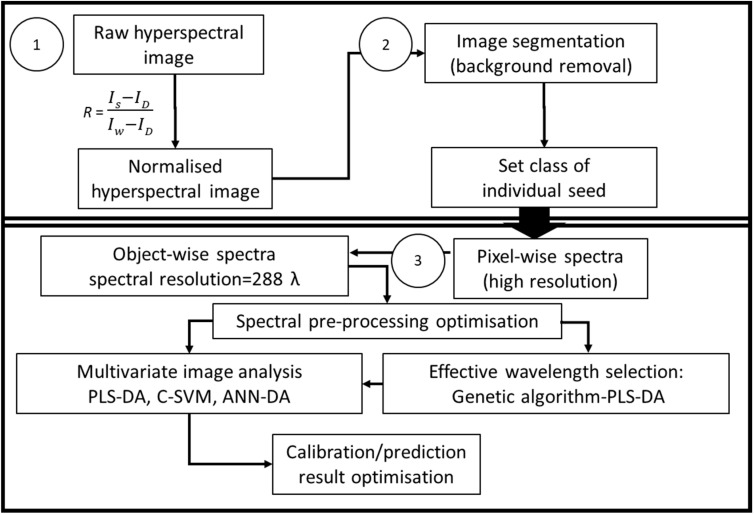
Flow chart of steps for analysing raw hyperspectral image of seeds. (1) Normalisation of image based on white and dark reference; (2) Image segmentation for background removal; (3) Spectral pre-processing and classification model development.

A flow chart summarizing the image processing steps is shown in Figure 1.

#### 2.4.1. Data Extraction

The raw hyperspectral images acquired using NIR-HSI were corrected with white and dark reference images to obtain reflectance values using MATLAB (The Math-Works Inc., Natick, MA, USA, Version v.R2022). The corrected sample reflectance spectra were processed in MIA_Toolbox (v. 9.0 (2022), Eigenvector Research Inc., Manson, WA, USA). A reverse-mask was used for image segmentation by setting a numerical value of all background pixels to 0 and all pixels within the sample to 1. An averaged spectrum was generated for each seed sample to perform object-wise spectral pre-processing and analysis optimization.

#### 2.4.2. Pre-Processing and Multivariate Analysis Methods

Prior to multivariate analyses, the original NIR spectra were pre-processed to remove noise interference and irrelevant information, thereby improving the robustness of the model and improving the signal-to-noise. Based on PLS-DA modelling, optimal pre-treatment was determined for endophyte and WE discrimination using methods including detrend, second order automatic weighted least squares (baseline, order = 2), mean centring, first-order derivative, extended multiplicative scatter correction/signal correction (EMSC), standard normal variate (SNV) and orthogonal signal correction (OSC).

PLS-DA is a supervised multivariate analysis used to reduce data dimensionality. It is a widely used method in NIR-HSI spectral analysis and is a variant of the linear regression model, partial least squares (PLS), that can be used when the response variable (Y) is categorical [22]. In this study, it was used to optimize the separation between groups by linking the X (spectral data) and Y as a set of class labels (perennial ryegrass cultivars or endophyte presence/absence) using a latent variable approach to model their maximum covariance structures.

For the SVM and ANN-DA models, PLS data compression was used to reduce dimensionality and overfitting. The SVM algorithm is also used as a supervised statistical method for high dimensional data but has the ability to solve non-linear problems based on the maximum margin between the boundaries of groups called the hyperplane [23]. In the present study, the use of radial basis function (RBF) was used as a kernel with optimization of an adjustable cost function (C) and gamma (δ)-, which indicated how strongly misclassifications should be penalized and thus, henceforth referred to as C-SVM. ANN-DA is also a non-linear algorithm based on a collection of connected nodes referred to as artificial neurons. Herein, the number of nodes in the first hidden layer was optimized to 10.

#### 2.4.3. Model Evaluation

The models were evaluated using true positive rate (sensitivity) (Equation (2)) using the metrics *TP* (true positive) and *FN* (false negative) and true negative rate (specificity) (Equation (3)), which uses the metrics *TN* (true negative) and *FP* (false positive). The true positive rate (sensitivity) and true negative rate (specificity) are used to determine the classification error (*CE*) (Equation (4)), which is equivalent to the average of the false positive rate and false negative rate.

(2) $$sensitivity=\frac{TP}{TP+FN}$$

(3) $$specificity=\frac{TN}{TN+FP}$$

(4) CE=1-(sensitivity+specificity)/2

(5) Overall accuracy (%) = 100 − CE

Cross validation (CV) was performed for all models, using Venetian blinds by applying 10 data splits, with one sample per blind used to assess individual models and to select the optimum number of LVs in PLS-DA. The dataset was organized in class order by cultivar. A total of 288 averaged spectra for each seed image was acquired for 1249 seeds, which were assigned a class based on endophyte/WE or cultivar. The prediction dataset was generated by using a Kennard-Stone algorithm, which selects a subset of samples that uniformly cover the dataset and includes exterior samples as the calibration set (used to develop the model), and the remainder is placed in the test set (to assess the model). The dataset was split into 95% calibration and 5% prediction. Overall accuracy (Equation (5)) was based on a class error of prediction or cross validation, whichever was higher within the classes.

#### 2.4.4. Genetic Algorithm

A genetic algorithm (GA) optimiser, using PLS-DA as a classifier (GA-PLS-DA), was used to define the most effective wavebands (Table 1). These wavebands were then used as above for the classification model.

## 3. Results and Discussion

To discriminate E+ (endophyte strain NEA12) from E− (without endophyte, WE), irrespective of perennial ryegrass cultivar and seed batch, classification models were generated based on optimised pre-processed spectra (Table 2). These same pre-processed spectral data and optimised pre-processing steps were subsequently used to discriminate perennial ryegrass cultivars within the same cohort. 

### 3.1. Endophyte Discrimination—Pre-Treatment

Spectral pre-processing entails algorithms that correct noise and artifacts generated from light scattering and variation in surface morphology. This step is important in developing a robust model. Smoothing can contribute to the removal of instrumental noise without reducing spectral resolution.

The calibration set was composed of 1005 samples for E+ (*n* = 551) and E− (*n* = 454), and the model was verified with a validation set of 52 samples [E+ (*n* = 26) and E− (*n* = 26)], after the establishment of the PLS-DA model. The pre-processing method utilised includes detrending, weighted least squares baseline algorithm (baseline), orthogonal signal correction (OSC) and mean centring. The baseline pre-treatment iteratively performs baseline fitting to each spectrum and determines the variables above the baseline while limiting the negative signals. Mean centring calculates the mean of each column (variable) and subtracts this from the column. It indicates the deviation from the spectral mean for each row (seed) from the original data matrix. The additional pre-treatment methods that were trialled include orthogonal signal correction (OSC), extended multiplicative scatter correction/signal correction (EMSC) and derivatives. OSC achieves the removal of excessive background by filtering from the spectral matrix X, the component that is not correlated to Y, i.e., it removes the uninformative component from the response variable Y [24,25]. EMSC removes undesirable scatter effects from the data matrix and performs polynomial reference correction and baseline fitting to the wavelength axis prior to data modelling [26]. Derivatives (mainly first and second derivatives) are methods used to remove additive and/or multiplicative effects in spectral data. The first derivative removes baseline drifts, and the second derivative has the function of resolving linear trends and sharpening spectral features.

It is important to evaluate the effect of various pre-processing algorithms on the final model. The results of calibration, validation and prediction for the PLS-DA models using selected pre-treatments to discriminate E+ or E− seeds are presented in Table 1. A combination of detrend, baseline, derivative, EMSC, OSC and mean centre (Table 1, pre-treatment no.6) yielded the lowest prediction (CEP = 3.8%) and cross validation errors (CV = 11.0%) and the lowest number of latent variables (LV = 4), indicating a more parsimonious model with an 89% overall accuracy. Removing EMSC from the pre-treatment resulted in a higher CEP (9.6%) and marginally higher CV error (11.5%) with an increase in the number of LVs. The best prediction of the E+/E− discrimination was achieved with the inclusion of OSC. The optimised pre-processing steps, as shown in Table 1, were used to evaluate the performance of models henceforth.

### 3.2. Differentiation of E+ (NEA12) and E− (WE) Seeds

#### 3.2.1. Discriminant Models for E+ and E− Using Full Spectra

**Figure 2:**
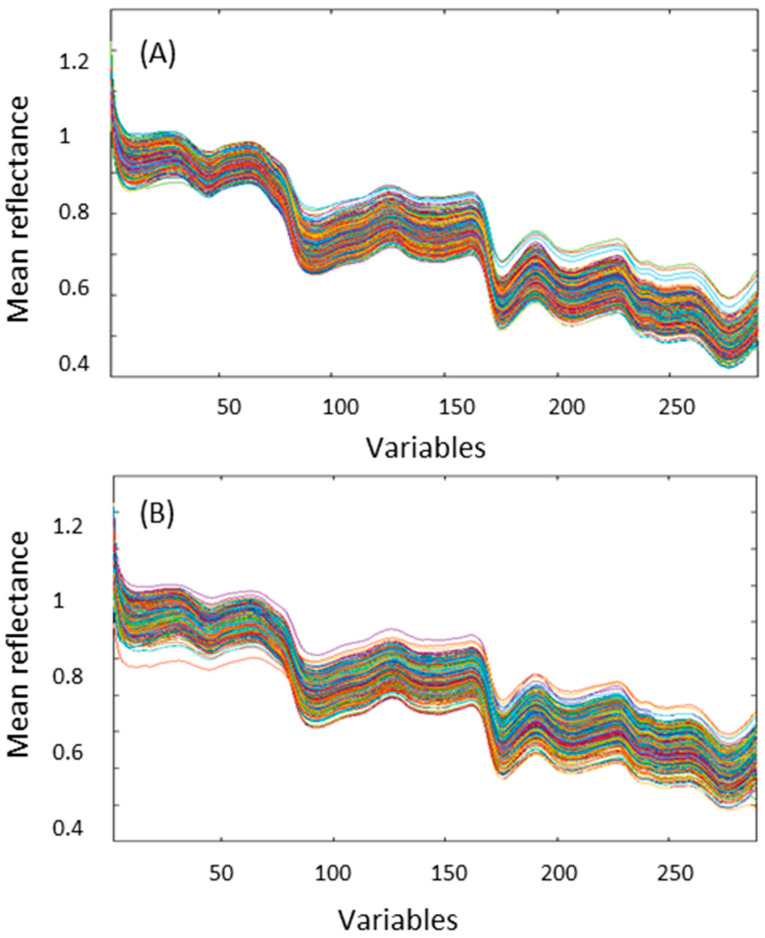
Raw NIR spectra of E− (WE) seed (**A**) and E+ (NEA12 endophyte-infected) seed (**B**). The variables represent 288 wavelengths acquired in the spectral range (1000–2500 nm).

**Figure 3:**
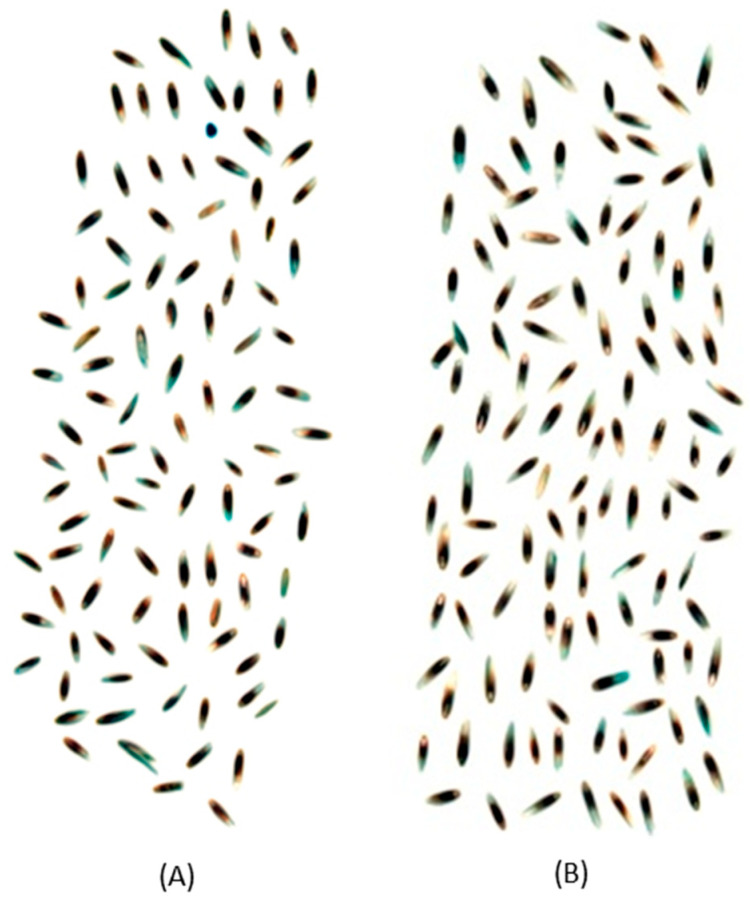
True hyperspectral image of Trojan- E− (WE) seed (**A**) and Trojan- E+ (NEA12 endophyte-infected) seed (**B**).

It is known that the resident endophyte alters the physiology of the host seed [27,28,29,30]. For example, metabolite composition associated with sugars (i.e., mannitol, ribitol and trehalose), antioxidants (i.e., tocochromanols and glutathione) and alkaloid content (peramine, ergovaline and lolitrem B) are altered in *Epichloë* endophyte-infected seed, which may account for the wider absorbance range in the spectra of E+ compared to E− seed (Figure 2). Perennial ryegrass seeds are small in length (5 to 8 mm) and width (1 to 1.5 mm (midpoint)) compared to grain crops such as maize and wheat and thus discerning physiological traits may not be as obvious due to constraints in not only size but quantities of individual seed required for evaluation in a high-throughput manner. The presence of the naturally occurring beneficial fungal symbiont also contributes to the complexity of the seed. An example of a true hyperspectral image of WE and NEA12 perennial ryegrass seed of the cultivar Trojan is shown in Figure 3. 

The results of calibration, validation, and prediction models to discriminate E+ and E− are shown in Table 3. Using the optimised pre-treatments (Table 2, pre-treatment no. 6), the PLS-DA (CEP = 3.8%; CV = 11.0%) model outperformed ANN-DA (CEP = 5.8%; CV = 11.0%) and C-SVM (CEP = 19.2%; CV = 23.7%) models resulting in an 89% overall accuracy.

While there are many applications that are developed to identify microbial pathogens that sometimes result in visual defects, to our knowledge, this is the first NIR-HSI method to accurately detect a beneficial, asymptomatic, fungal endophyte in the seed. Comparable studies reported are for the detection of early stages of fungal infection/disease or identification of atoxicogenic fungal strains [12,16,31,32].

Senthilkumar et al. [12] reported differences in sterile and infected kernels of stored barley on statistical classifers, including linear, quadratic and Mahalanobis, using NIR-HSI wavelengths 1000–1600 nm. The images were acquired every 2 weeks and showed at least 80% classification accuracy at initial periods of fungal infection and 100% after four weeks of infection for all classifiers. Based on fluorescene hyperspectral imaging, Yao et al. [31] reported 100% classification accuracy of atoxicogenic and toxiogenic *Aspergillus flavus* fungal strains in maize kernels based on an LDA classifier. The kernels were on the germ side and adjacent to the infected kernels, identifiable by their bright fluorescence. Fluorosence imaging is based on a visible near-infrared (VIS-NIR) hyperspectral imager covering the 400–700 nm range, thus having similar spectral and spatial resolutions. A classification accuracy of 94.4% of healthy and contaminated corn kernels (germ side) was also achieved based on aflatoxin threshold levels of 20 ppb and 100 ppb, respectively. Lee et al. [16] described the identification of bacteria-infected watermelon seed using NIR-HSI with a spectral range of 400–1000 nm using PLS-DA and least squares support vector machine (LS-SVM) with a classification accuracy of 91.7% and 90.5% respectively. Although these studies did not pertain to endophyte-infected seeds, we surmise that by applying statistical classifiers on hyperspectral imaging acquistion data, low levels of contamination and early stages of fungal and bacterial infection can be detected in a seed.

#### 3.2.2. Discriminant Models for E+ and E− Using Effective Wavelengths

Identification of effective wavelengths (EW) removes redundancy and collinearity of spectral data and allows the use of low-cost multispectral cameras in place of hyperspectral cameras. It also increases the data-processing speed and reduces the need for high-end computing infrastructure, processing power and storage. There are many variable selection methods for NIR-HSI spectral data. In this study, Genetic Algorithm (GA) was used to select optimal wavelengths, along with PLS-DA as a classifier for effective waveband selection. GA is inspired by natural evolution and natural genetics that is used to select the most effective waveband combination based on the “survival of the fittest” approach [33], where a string of ‘chromosomes’ or individuals (i.e., a subset of wavebands) are assigned a fitness score using root mean standard error for cross-validation (RMSECV) fitness function, based on how well the individual performs in distinguishing between treatments. This is evolved over successive generations, using waveband selection (wavebands with high fitness scores), mutation (modification of individual by the introduction of new wavebands) and crossover (random selection of wavebands from two individuals) to produce waveband combinations that produce the lowest RMSECV scores- a better fit to the data, in GA-PLS-DA modelling.

The number of wavebands used for classification was reduced to 75 from 288 using the GA-PLS-DA model (Table 4). The 75 wavelengths are the most effective wavelength combination selected. A combination of detrend, baseline, derivative, OSC and mean centre (Table 2, pre-treatment no. 5) yielded the lowest calibration, validation and prediction errors for the PLS-DA (CEP = 11.4%; CV = 11.7%; LV = 9) model compared to ANN-DA, which resulted in consistent prediction errors (CEP = 11.4%) but a slightly higher cross validation error (CV = 12.2%). The C-SVM model resulted in poor prediction (CEP = 15.6%) and cross validation errors (CV = 13.3%). Although the PLS-DA model performed the best, it was not unexpected that the cross validation and prediction errors were moderately higher than full wavelength classification models, but the minor compromise to the overall accuracy (88.3%) was acceptable.

Wallays et al. [34] described the use of GA-PLS-DA for the selection of wavebands in the range of 400 to 950 nm of NIR-HSI acquisition data for discrimination between pure kernels and material other than grain (MOG) comprising chaff and straw of diverse varieties of wheat. Seo et al. [35] also describes the use of NIR-HSI (950–2500 nm) to identify cucumber green mosaic virus in watermelon seed with classifiers including PLS-DA, SVM and LS-SVM, showing 78%, 81.3% and 92.3% classification accuracy, respectively, based on healthy vs. infected seed. GA-PLS-DA implementation resulted in 93.4% accuracy of selected bands using the LS-SVM classifier.

These studies indicate that GA-PLS-DA is an effective technique for the selection of wavebands and reduce redundancy in hyperspectral images for the identification of various traits in small seed and grain samples. In this study, hyperspectral waveband selection could allow for the subsequent development of low-cost multispectral sensors for online measurement.

### 3.3. Differentiation of Perennial Ryegrass Cultivars

#### 3.3.1. Discriminant Models for Perennial Ryegrass Cultivars Using Full Spectra

**Figure 4:**
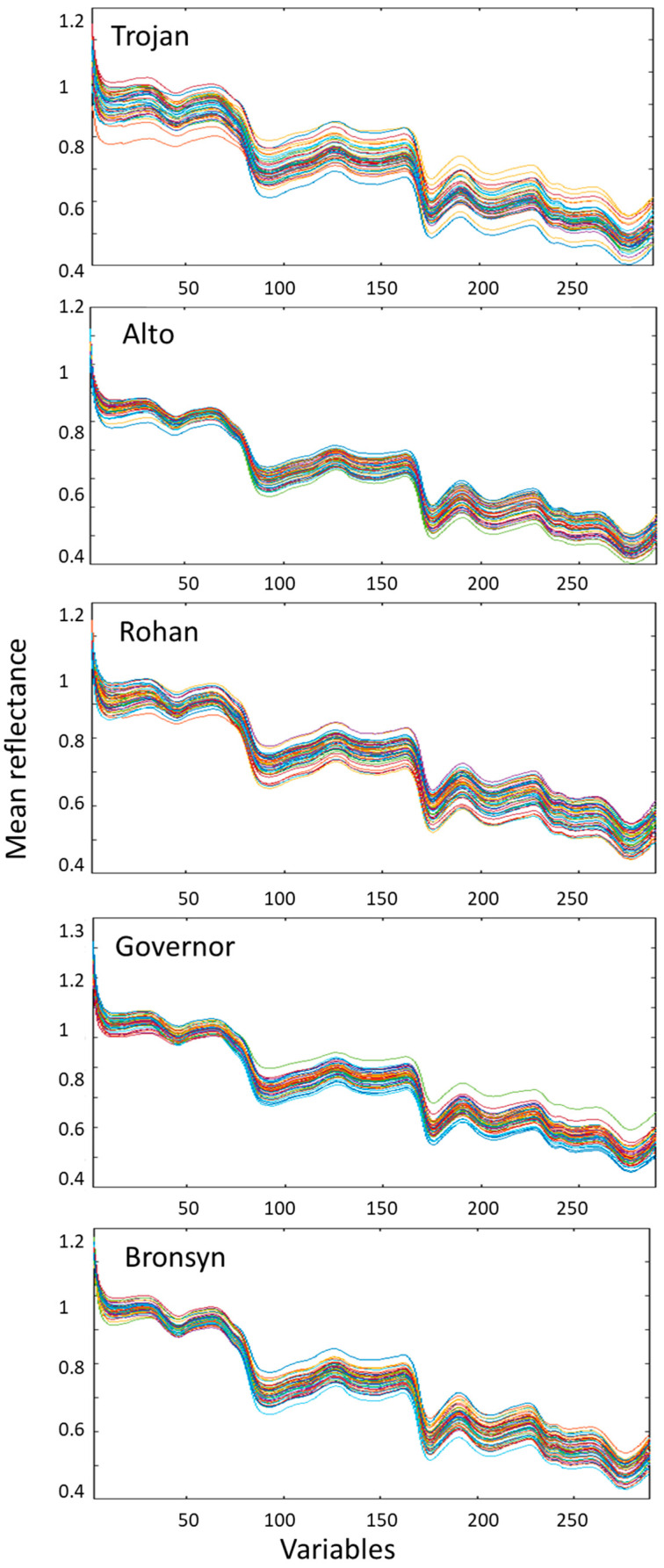
Raw NIR spectra of Trojan, Alto, Rohan, Governor and Bronsyn. The variables represent 288 wavelengths acquired in the spectral range (1000–2500 nm).

The raw NIR spectral data plot of 5 perennial ryegrass cultivars—Trojan, Alto, Rohan, Governor and Bronsyn—is shown in Figure 4. The results of calibration, validation, and prediction models to discriminate cultivars are shown in Table 5 using a combination of pre-treatments, including detrend, baseline, derivative, EMSC, OSC and mean centre (Table 2, pre-treatment no. 6). Overall, the ANN-DA model performed moderately better than PLS-DA and C-SVM, particularly in the class error prediction for all cultivars. With exception to cross validation error of Governor (CV = 6.9%), individual cultivars in the ANN-DA model revealed lower errors compared to PLS-DA and C-SVM with a 90% overall accuracy.

#### 3.3.2. Discriminant Models for Perennial Ryegrass Cultivars Using Effective Wavelengths

The number of wavebands used for classification was reduced to 87 from 288 using the GA-PLS-DA model. The 87 wavelengths are the most effective wavelength combination selected using a combination of detrend, baseline, derivative, OSC and mean centre pre-processing (Table 2, pre-treatment no. 5). The results of calibration, validation and prediction are shown in Table 6. Overall, the C-SVM model revealed a lower rate for the cross validation in Trojan (CV = 9.3%), Rohan (CV = 10.0%) and Governor (CV= 7.6%) and prediction errors in Alto (CEP = 1.1%), Governor (CEP = 0.0%) and Bronsyn (CEP = 0.0%) compared to PLS-DA and ANN-DA. 

The overall performance of the ANN-DA model with full spectra (288 wavebands) compared to model performance using 87 selected wavebands based on C-SVM shows that there is no extensive compromise in the overall accuracy of 89.1%.

Cultivar identification performed in other seed applications has also shown 80–100% levels of calibration and prediction accuracy [6]. A study that discriminated between rice seed cultivars of the same age to avoid batch effects using NIR-HSI covering wavelengths 874–1734 nm resulted in 80% classification accuracy using PLS-DA and KNN and 100% accuracy using Soft Independent Modeling of Class Analogy (SIMCA), RF and SVM classifiers. Wavebands were selected using weighted regression coefficients of the PLS-DA and resulted in over 80% classification rates [19]. Similar results were achieved by Wu et al. in oat seed varieties of the same age, which yielded 99.19% accuracy in the validation set using deep convolutional neural network (DCNN) on NIR-HSI (875–1734 nm) acquisition data [36]. The classification accuracies of traditional classifiers LR (98.69%), SVM (98.05%) and linear SVM (97.88%) improved when combined with DCNN, resulting in 98.72, 99.05 and 99.02%, respectively. Wavebands were selected based on the variable selection using the second derivative method, and SVM (87.31%) performed better than linear SVM (84.21%) and LR (84.92%).

The classification accuracies of previous studies are comparable to the current study for the varietal classification of seeds, indicating traditional classifiers and deep learning algorithms are effective statistical methods for discrimination. It is noteworthy that in the present study, at least 90% and 89.1% classification accuracy were achieved based on full wavelengths and effective wavelengths, respectively, of perennial ryegrass seed cultivars. The minor reduction in classification accuracy based on EW is similar to some of the previous studies described and is likely due to a reduction in collinearity between wavebands. Moreover, the elimination of effects from seed age and batch in the multivariate models developed in this study indicates the potential to identify other cultivars and associated endophytes of perennial ryegrass seed of any age and batch. 

## 4. Conclusions

The study investigated non-destructive, rapid, seed-based diagnostics of perennial ryegrass using NIR-HSI technology. E+/E− seed from five different cultivars and of varying ages was effectively discriminated. Accurate cultivar discrimination was achieved using five cultivars (Trojan, Alto, Rohan, Bronsyn and Governor) of perennial ryegrass that also varied in endophyte status and age of the seed. A PLS-DA, C-SVM and ANN-DA model was developed using averaged NIR spectra for each seed, encompassing 288 discrete wavelengths using optimal pre-treatment algorithms developed on a PLS-DA model. The best discrimination accuracy for E+/E− was the PLS-DA model, which yielded an 89% overall accuracy. Effective wavelength selection using GA-PLS-DA on the NIR spectral matrix for classes E+/E− reduced wavebands to 75 and resulted in a minor compromise to the overall accuracy (88.3%) based on PLS-DA. Classification models were generated on these same spectra for cultivars (Trojan, Alto, Rohan, Bronsyn and Governor). The ANN-DA model performed better than C-SVM and PLS-DA, with a 90% overall accuracy. Effective wavelength selection using GA-PLS-DA reduced the wavebands to 87, and the C-SVM model performed the best, with no extensive compromise in model performance, revealing an 89.1% overall accuracy.

Thus, hyperspectral-NIR reflectance imaging is promising for the discrimination of seed with endophyte strain NEA12 from seed without endophyte as well as cultivars (Trojan, Alto, Rohan, Bronsyn and Governor). The established imaging pipeline has not only the potential to be applied to other perennial ryegrass cultivars and endophytes but other pasture grass species, such as tall fescue and its associated endophytes. The limited loss of accuracies in using effective wavelengths also demonstrates practical deployment using low-cost multispectral sensors for single seed analysis.

## Figures and Tables

**Table 1:** Genetic algorithm parameters.

Parameters	
Population	64
Max Generations	100
Crossover	Double
Mutation rate	0.005
% Convergence	50
Number of GA Iterations	1

**Table 2:** Pre-treatment method optimization for PLS-DA classification models using full spectra for E+ (*n* = 551) and E− (*n* = 454) calibration dataset and E+ (*n* = 26) and E− (*n* = 26) prediction dataset.

No.	Pre-Treatment	Endophyte Status	Sensitivity (Cal)	Class Error (Cal)	Sensitivity (CV)	Class Error (CV)	Sensitivity (Pred)	Class Error (Pred)
1	Detrend, Baseline (order = 2); Mean centre (LV = 19)	E+	0.904	9.9%	0.853	13.7%	0.846	9.6%
		E−	0.899		0.872		0.962	
2	Detrend, Baseline (order = 2), 2nd Derivative (order = 2, 15 pt), Mean centre (LV = 19)	E+	0.906	10.1%	0.851	14.8%	0.846	15.4%
		E−	0.892		0.852		0.846	
3	Detrend, Baseline (order = 2), 2nd Derivative (order = 2, 15 pt), EMSC, Mean centre (LV = 18)	E+	0.900	10.6%	0.858	14.6%	0.885	9.6%
		E−	0.888		0.850		0.923	
4	Detrend, Baseline (order = 2), OSC, Mean centre (LV = 11)	E+	0.966	4.0%	0.887	10.4%	0.885	11.5%
		E−	0.954		0.905		0.885	
5	Detrend, Baseline (order = 2), 2nd Derivative (order = 2, 15 pt), OSC, Mean centre (LV = 6)	E+	0.962	4.2%	0.882	11.5%	0.962	9.6%
		E−	0.954		0.888		0.846	
6	Detrend, Baseline (order = 2), 2nd Derivative (order = 2, 15 pt), EMSC, OSC, Mean centre (LV = 4)	E+	0.956	4.1%	0.893	11.0%	0.962	3.8%
		E−	0.963		0.888		0.962	

**Table 3:** Classification models using full spectra for E+ (*n* = 551) and E− (*n* = 454) calibration dataset and E+ (*n* = 26) and E− (*n* = 26) prediction dataset.

Model	Endophyte Status	Sensitivity (Cal)	Class Error (Cal)	Sensitivity (CV)	Class Error (CV)	Sensitivity (Pred)	Class Error (Pred)
PLS-DA	E+	0.956	4.1%	0.893	11.0%	0.962	3.8%
LV = 4	E−	0.963		0.888		0.962	
C-SVM *	E+	0.873	20.4%	0.849	23.7%	0.731	19.2%
C = 1	E−	0.718		0.676		0.885	
δ = 0.32							
ANN-DA *	E+	0.971	2.5%	0.895	11.0%	0.962	5.8%
	E−	0.974		0.885		0.923	

* partial least squares data compression performed on the x-block based on 4 latent variables.

**Table 4:** Classification models using 75 optimal wavelengths for E+ (*n* = 550) and E− (*n* = 455) calibration dataset and E+ (*n* = 27) and E− (*n* = 25) prediction dataset.

Model	Endophyte Status	Sensitivity (Cal)	Class Error (Cal)	Sensitivity (CV)	Class Error (CV)	Sensitivity (Pred)	Class Error (Pred)
PLS-DA	E+	0.922	9.6%	0.898	11.7%	0.852	11.4%
LV =9	E−	0.886		0.868		0.920	
C-SVM *	E+	0.916	10.3%	0.887	13.3%	0.889	15.6%
C = 100	E−	0.877		0.846		0.800	
δ = 0.003							
ANN-DA *	E+	0.929	6.8%	0.870	12.2%	0.852	11.4%
	E−	0.899		0.885		0.920	

* partial least squares data compression performed on the x-block based on 9 latent variables.

**Table 5:** Classification models using full spectra for cultivars Trojan (*n* = 369); Alto (*n* = 86); Rohan (*n* = 277); Governor (*n* = 181) and Bronsyn (*n* = 92) calibration dataset and Trojan (*n* = 16); Alto (*n* = 10); Rohan (*n* = 11); Governor (*n* = 11); Bronsyn (*n* = 4) prediction dataset.

Model	Cultivar	Sensitivity (Cal)	Class Error (Cal)	Sensitivity (CV)	Class Error (CV)	Sensitivity (Pred)	Class Error (Pred) (CV)
PLS-DA	Trojan	0.957	4.1%	0.900	9.5%	1.000	0.0%
LV = 18	Alto	0.965	4.7%	0.919	7.7%	1.000	3.6%
	Rohan	0.921	7.6%	0.881	10.8%	0.909	4.5%
	Governor	0.967	4.1%	0.950	5.8%	1.000	4.9%
	Bronsyn	0.957	4.0%	0.902	7.7%	1.000	2.1%
C-SVM *	Trojan	0.981	1.9%	0.900	8.6%	1.000	2.8%
C = 10	Alto	0.919	4.2%	0.767	12.3%	0.900	6.2%
δ = 0.01	Rohan	0.957	2.6%	0.856	10.2%	0.727	13.6%
	Governor	0.989	1.0%	0.878	7.2%	0.909	7.0%
	Bronsyn	0.989	0.6%	0.913	4.8%	0.750	13.5%
ANN-DA *	Trojan	0.992	2.5%	0.892	8.4%	1.000	0.0%
	Alto	1.000	2.8%	0.919	6.3%	1.000	1.2%
	Rohan	1.000	2.0%	0.874	10.0%	0.909	4.5%
	Governor	0.994	3.1%	0.906	6.9%	1.000	2.4%
	Bronsyn	0.989	1.3%	0.902	7.0%	1.000	0.0%

* partial least squares data compression performed on the x-block based on 18 latent variables.

**Table 6:** Classification models using 87 optimal wavebands for cultivars Trojan (*n* = 366); Alto (*n* = 91); Rohan (*n* = 271); Governor (*n* = 185) and Bronsyn (*n* = 92) calibration dataset and Trojan (*n* = 19); Alto (*n* = 5); Rohan (*n* = 17); Governor (*n* = 7) and Bronsyn (*n* = 4) prediction dataset.

Model	Cultivar	Sensitivity (Cal)	Class Error (Cal)	Sensitivity (CV)	Class Error (CV)	Sensitivity (Pred)	Class Error (Pred) (CV)
PLS-DA	Trojan	0.932	8.0%	0.896	10.9%	0.895	9.8%
LV = 19	Alto	0.956	4.1%	0.923	5.8%	1.000	5.3%
	Rohan	0.889	11.3%	0.878	12.4%	0.941	7.2%
	Governor	0.903	8.1%	0.886	9.7%	1.000	11.1%
	Bronsyn	0.946	6.4%	0.913	8.3%	1.000	1.0%
C-SVM *	Trojan	0.940	5.0%	0.904	9.3%	0.842	10.9%
C = 1	Alto	0.967	1.8%	0.857	7.5%	1.000	1.1%
δ = 0.03	Rohan	0.915	5.8%	0.852	10.0%	0.882	8.7%
	Governor	0.951	3.0%	0.870	7.6%	1.000	0.0%
	Bronsyn	0.913	4.6%	0.826	9.0%	1.000	0.0%
ANN-DA *	Trojan	0.956	4.1%	0.888	10.1%	0.789	13.6%
	Alto	0.967	2.2%	0.934	4.8%	1.000	2.1%
	Rohan	0.934	5.5%	0.860	11.5%	0.941	11.5%
	Governor	0.919	6.2%	0.886	8.8%	1.000	1.1%
	Bronsyn	0.957	3.7%	0.902	7.4%	1.000	1.0%

* partial least squares data compression performed on the x-block based on 19 latent variables.

## Data Availability

Data will be provided on reasonable request.
